# Understanding Cardio-Oncology: A Survey-Based Study Conducted by the Heart Failure Association of the Polish Cardiac Society and the Polish Society of Clinical Oncology

**DOI:** 10.3390/jcm15031240

**Published:** 2026-02-04

**Authors:** Sebastian Szmit, Jarosław Kępski, Marcin Książczyk, Maciej Krzakowski, Małgorzata Lelonek

**Affiliations:** 1 Department of Cardio-Oncology, Centre of Postgraduate Medical Education, 01-813 Warsaw, Poland; 2 Cardio-Oncology Unit, Maria Sklodowska-Curie National Research Institute of Oncology, 02-781 Warsaw, Poland; 3 Department of Noninvasive Cardiology, Medical University of Lodz, 90-549 Lodz, Poland; 4 Department of Lung Cancer and Thoracic Tumours, Maria Sklodowska-Curie National Research Institute of Oncology, 02-781 Warsaw, Poland; maciej.krzakowski@nio.gov.pl

**Keywords:** cardio-oncology, guidelines, heart failure, venous thromboembolism, lung cancer, cardiologists, oncologists

## Abstract

**Background**: The European Society of Cardiology (ESC) published the first guidelines on cardio-oncology in 2022. Implementing the 272 proposed recommendations into everyday clinical practice has become a mandatory challenge for countries belonging to the ESC community. **Methods**: The study aimed to assess cardio-oncology knowledge and the degree of implementation of ESC guidelines among cardiologists registered with the Heart Failure Association of the Polish Cardiac Society and oncologists from the Polish Society of Clinical Oncology. Physicians were invited via email and voluntarily chose to participate by completing a 20-question questionnaire. **Results**: Among the 104 respondents, half (50%) were cardiologists, and the majority (80%) had more than ten years of clinical experience. A total of 38.8% of specialists practiced outpatient medicine, while 41.7% worked in academic centres. The majority (58.3%) consult fewer than ten cardio-oncology patients per week, with less than 8% of specialists having the greatest experience (>25 consultations per week). Most physicians were familiar with the ESC guidelines on cardio-oncology. Cardiologists more frequently indicated heart failure as the main problem in cancer patients (OR = 5.82; 95% CI: 2.08–16.22; *p* = 0.0007), ordered echocardiography and ECG together with cardiovascular risk factors control (OR = 4.01; 95% CI: 1.74–9.25; *p* = 0.001) during long-term follow-up, chose angiotensin converting enzyme inhibitor or angiotensin receptor blocker (ACEi/ARB) combined with calcium channel blocker (CCB) for treating hypertension (OR = 3.9; 95% CI: 1.56–9.75; *p* = 0.003), and rarely monitored lipid profile based on the type of cancer therapy (OR = 0.09; 95% CI: 0.03–0.26; *p* = 0.000009). Oncologists more often observed cardiovascular issues in lung cancer (OR = 3.78; 95% CI: 1.58–9.05; *p* = 0.002), recognized venous thromboembolism as the most common problem in cardio-oncology (OR = 6.52; 95% CI: 2.7–15.73; *p* = 0.00002), opted for ACEI/ARB monotherapy in the management of high blood pressure (OR = 11.76; 95% CI: 2.49–55.54; *p* = 0.002), and significantly more often chose low-molecular-weight heparin in the treatment of asymptomatic incidental pulmonary embolism (OR = 5.93; 95% CI: 2.47–14.24; *p* = 0.00006). **Conclusions**: The understanding of cardio-oncology varies significantly between cardiologists and oncologists. Although the survey was conducted only in one country (Poland), its results may serve as a reference point for structural reforms with building implementation strategies of ESC guidelines in daily practice in other countries.

## 1. Introduction

The introduction of modern therapies to oncology has significantly improved overall survival times, even in metastatic cancers. The best example is that immune checkpoint inhibitors have resulted in a 5-year survival rate of approximately 50% in metastatic melanoma or renal cell carcinoma [1]. However, this trend is sometimes limited by cardiovascular side effects that may contribute to treatment discontinuation and premature death. The best example is breast cancer, where during adjuvant treatment, discontinuation of therapy for cardiological reasons affects approximately 15% of patients and results in a significant worsening of the prognosis [2].

New cardiovascular issues or destabilization of previously well-controlled cardiovascular diseases are observed in patients treated with anticancer drugs. Therefore, in cardio-oncology it is important to properly define both permissive cardiotoxicity and toxicity requiring discontinuation of cancer therapy [3]. Patients with newly diagnosed cancer also have a high prevalence of concomitant cardiovascular disease. Available data confirmed that coronary artery disease, carotid artery disease, peripheral vascular disease, cerebrovascular disease, or heart failure could be diagnosed in 33% patients with hematologic malignancies, 43% with lung cancer, 17% with breast cancer, 26% with colon cancer, 35% with renal cell carcinoma, and 26% with head and neck cancers [4]. Cardiovascular co-morbidities can be a barrier to optimal cancer therapy.

When conducting an echocardiography examination before starting anticancer treatment, significant abnormalities in heart function can often be detected. In hemato-oncology the most frequently detected echocardiographic abnormality was increased high left atrial volume index (39%), diastolic dysfunction 2nd or 3rd degree (15.5%), elevated tricuspid regurgitation velocity (14.4%), increased E/e’ (6.5%) and decreased left ventricular systolic dysfunction (3.6%) [5]. In addition, cardiovascular diseases, such as heart failure, may be a risk factor for cancer [6,7]. It can be concluded that the estimated adjusted hazard ratio for cancer in patients with heart failure may be even 1.46–1.71, but not all studies confirmed such significant increased risk [8]. Therefore, the relationship between oncology and cardiology is complex and multifaceted.

The International Cardio-Oncology Society (IC-OS) proposed the new comprehensive definition of cancer therapy-related cardiovascular toxicity (CTR-CVT), which includes any cardiovascular disorder associated with anticancer treatment [9]. Cancer therapy-related cardiac dysfunction (CTRCD) is one of the main problems.

Cardio-oncology was born from the need for highly specialized cardiology care for cancer patients [10]. This gave rise to the specialization of cardio-oncologists who integrate knowledge of cardiology and oncology in clinical practice [11].

In 2022, the European Society of Cardiology (ESC) published the first guidelines on cardio-oncology [12]. The document presents extensive, comprehensive knowledge in oncology and cardiology, including the effects of anticancer therapies and their impact on the circulatory system, as well as practical recommendations for everyday clinical practice. The implementation of these recommendations with an appropriate quality has become the primary challenge for physicians caring for cancer patients with cardiovascular disorders [13].

###  Objective

The main goals of the study were to assess the perception of needs and challenges in cardio-oncology and the feasibility of implementing the ESC guidelines among cardiologists affiliated with the Heart Failure Association of the Polish Cardiac Society and oncologists from the Polish Society of Clinical Oncology.

## 2. Materials and Methods

The survey questions were prepared by the Chair of the Heart Failure Association of the Polish Cardiac Society (Małgorzata Lelonek), President of the Polish Society of Clinical Oncology (Maciej Krzakowski), one of the authors of the ESC guidelines on cardio-oncology (Sebastian Szmit), and two young scientists (Jarosław Kępski and Marcin Książczyk as representatives of the generation of young physicians shaping the future of cardio-oncology). The authors of the questionnaire developed it based on their own daily clinical observations. Direct inspiration came from discussions during scientific congresses and hospital meetings regarding the published European Society of Cardiology guidelines on cardio-oncology [12]. These discussions focused on the usefulness of the guidelines for the daily work of cardiologists and oncologists. Many cardiologists were surprised by the amount of new knowledge they gained. Oncologists, however, were seeking information on how the guidelines would actually impact their daily decisions regarding cancer treatment. It seemed appropriate to compare the understanding of the guidelines by oncologists and cardiologists.

The survey was anonymous, voluntary and conducted electronically by sending emails to members of the Heart Failure Association of the Polish Cardiac Society and members of the Polish Society of Clinical Oncology. The survey included 20 single-choice questions. The first six questions characterized the responders’ specialties (the first question: oncologist vs. cardiologist) and experience in cardio-oncology (questions 2–6 and results presented in Table 1). The subsequent four questions examined the respondents’ understanding of the 2022 ESC Guidelines in clinical practice (results in Table 2). The following five questions addressed the responders’ awareness of general cardio-oncology (results in Table 3), while the final five questions focused on selected challenging therapeutic decisions in cardio-oncology (results in Table 4).

### Statistical Analysis

The obtained data were subjected to statistical analysis. Categorical variables were presented as percentages. Responses between cardiologists and oncologists were compared using Fisher’s and χ^2^ tests.

A univariate logistic regression model was used to evaluate odds ratios with confidence intervals to identify the combined relationships between medical specialities and main responses to certain questions. All analyses were made using StatSoft Statistica 13, and a *p*-value < 0.05 was deemed significant.

## 3. Results

### 3.1. Characteristics of Responders

The survey was completed by 52 (50%) cardiologists and 52 (50%) oncologists; in total, 104 questionnaires were completed. The first question was about specialty (oncologistvs. cardiologist), questions 2 to 6 (presented in Table 1) focused on length of medical practice, position in the medical centre, type and referral level of medical centre, and the average number of cardio-oncology patients consulted each week.

Based on questions 1 to 6, responders differed regarding their medical specialty, length of medical practice, position in the medical centre, type of medical centre and referral level, and the average number of cardio-oncology patients consulted each week. There were no significant differences in the characteristics of cardiologists and oncologists (Table 1).

**Table 1:** Comparison of characteristics of cardiologists and oncologists who responded to the survey.

Question	Response Options to the Question	Cardiologists *n* = 52	Oncologists *n* = 52 *	*p*-Value
How many * cardio-oncology patients do you consult/manage every week?	A. <10 patients	32 (61.5)	28 (54.9)	*p* = 0.29
	B. 10–25 patients	10 (19.2)	16 (31.4)	
	C. 26–50 patients	4 (7.7)	5 (9.8)	
	D. >50 patients	6 (11.5)	2 (3.9)	
Length of medical practice	A. <5 years	6 (11.5)	3 (5.8)	*p* = 0.24
	B. 5–10 years	7 (13.5)	5 (9.6)	
	C. 10–20 years	17 (32.7)	27 (51.9)	
	D. >20 years	22 (42.3)	17 (32.7)	
Position in the medical centre	A. doctor	38 (73.1)	43 (82.7)	*p* = 0.15
	B. Head of unit	7 (13.5)	8 (15.4)	
	C. coordinator	4 (7.7)	0	
	D. other	3 (5.8)	1 (1.9)	
Type of medical centre *	A. clinic	4 (7.7)	0	*p* = 0.2
	B. hospital	20 (38.5)	20 (39.2)	
	C. hospital and clinic	26 (50)	30 (58.8)	
	D. private sector	2 (3.9)	1 (2)	
Referral level of medical centre *	A. provincial	15 (28.9)	20 (39.2)	*p* = 0.68
	B. municipal	9 (17.3)	6 (11.8)	
	C. county	5 (9.6)	5 (9.8)	
	D. academic/university	23 (44.2)	20 (39.2)	

* one oncologist did not respond to three questions.

Responders had considerable experience in medical practice: 39 (37.5%) specialists had practiced for more than 20 years, 44 (42.3%) had worked between 10 and 20 years, and only 21 (20.2%) had practiced for less than 10 years. Nineteen (18.3%) participants held a managerial position.

The questionnaire was completed by 40 (38.8%) specialists who worked in inpatient clinics, 56 (54.4%) specialists who worked in both inpatient and outpatient clinics and 7 (6.8%) who worked only in outpatient clinics or private healthcare centres. Forty-three (41.7%) responders declared a practice in the academic centre.

Most of the respondents (60, 58.3%) consulted fewer than 10 cardio-oncologic patients per week, while 26 (25.2%) participants consulted between 10 and 25 patients weekly, 9 (8.7%) specialists consulted 26 to 50 patients weekly, and only 8 (7.8%) responders provided medical service to more than 50 patients each week.

### 3.2. Questions About the 2022 ESC Guidelines on Cardio-Oncology

The answers given by cardiologists and oncologists to four questions directly about ESC guidelines were compared in Table 2. There were significant differences in responses to the general question about the new cardio-oncology guidelines. Among cardiologists, the two most common responses were: “I read the guidelines and adhere to them” and “I read the guidelines but I think they are too complicated to adhere to them”—15 cardiologists (28.8%) provided these responses. Among oncologists, the most prevalent responses were: “I read the guidelines, but I think it will be difficult to adhere to them in Poland” (42 oncologists, 42.3%) and “I know the guidelines were published, but I did not read them yet” (17 oncologists, 32.7%).

**Table 2:** Comparison of answers given by cardiologists and oncologists about ESC guidelines on cardio-oncology.

Question	Response Options to the Question	Cardiologists *n* = 52	Oncologists *n* = 52	*p*-Value
2022 ESC Guidelines on cardio-oncology.	E. I know the guidelines were published but have not read them yet.	8 (15.4)	17 (32.7)	*p* = 0.005
	F. I read the guidelines and adhere to them.	15 (28.8)	9 (17.3)	
	G. I read the guidelines, but I think adhering to them in Poland won’t be easy.	14 (26.9)	22 (42.3)	
	H. I read the guidelines, but think they are too complicated to adhere to.	15 (28.8)	4 (7.7)	
According to the 2022 ESC Guidelines on cardio-oncology, cardiology assessment is recommended for oncology patients before, during, and after cancer treatments.	E. I do it in every patient in both inpatient and outpatient clinic.	27 (52.9)	11 (22)	*p* = 0.001
	F. I do it in every patient only in inpatient clinic.	11 (21.6)	15 (30)	
	G. I do it rarely.	1 (1.9)	11 (22)	
	H. I think it won’t be easy to do in Poland.	12 (23.5)	13 (26)	
2022 ESC Guidelines on cardio-oncology highlight the role of GLS in echocardiographic assessment.	E. I follow the GLS.	18 (38.3)	3 (6.5)	*p* = 0.00002
	F. I do not use GLS because of technical problems.	16 (34.0)	9 (19.6)	
	G. I do not know the rules of GLS use.	4 (8.5)	20 (43.5)	
	H. I follow the LVEF as I am not experienced enough to use GLS.	9 (19.2)	14 (30.4)	
2022 ESC Guidelines on cardio-oncology highlight the role of biomarkers in CV toxicity assessment during cancer treatment.	E. I use only NT-proBNP or BNP tests because of their availability.	1 (1.9)	3 (6)	*p* = 0.29
	F. I use only hs-cTnT tests because of their availability.	3 (5.8)	3 (6)	
	G. I use both NT-proBNP or BNP and hs-cTnT tests because of their availability.	46 (88.4)	38 (76)	
	H. I do not use the biomarkers tests.	2 (3.9)	6 (12)	

Legends: GLS—global longitudinal strain; LVEF—left ventricular ejection fraction; NT-proBNP—N-terminal pro-B-type natriuretic protein; BNP—B-type natriuretic peptide; hs-cTnT—high-sensitivity cardiac troponin T.

The second question was whether the guidelines recommend a cardiology assessment before, during and after cancer therapy. A significant proportion of cardiologists answered that they do this for every patient in inpatient and outpatient settings (27 cardiologists, 52.9%).

There were significant differences in responses to the third question about the role of global longitudinal strain (GLS) in cancer patients. The most common reactions among cardiologists were: “I follow the GLS (18, 38.3%)” and “I do not use GLS because of technical problems” (16, 34.0%). Oncologists, on the other hand, chose the other two response options: “I do not know the rules for GLS use” (20, 43.5%) and “I follow the LVEF as I am not experienced enough to use GLS” (14, 30.4%).

Interestingly, there were no differences in the perception of biomarkers’ role in assessing cardiotoxicity. The majority of cardiologists (46, 88.4%) and oncologists (38, 76%) responded as follows: “I use both N-terminal pro-B-type natriuretic protein (NT-proBNP) or B-type natriuretic peptide (BNP) and high-sensitivity cardiac troponin T (cTn Ths) tests because of their availability”.

### 3.3. General Understanding of Cardio-Oncology

The analysis of the establishment of cardio-oncology in daily practice, based on five questions and answers, is presented in Table 3. When asked about the type of cancer that causes the most cardiovascular complications, cardiologists gave significantly different answers compared to oncologists. Cardiologists most often indicated breast cancer (18, 36.7%), whereas oncologists referred to lung cancer (27, 55.1%). However, the responses of cardiologists and oncologists did not differ significantly when asked about the greatest diagnostic and therapeutic challenges they perceived. Cardiologists and oncologists similarly identified difficulties in the diagnosis and treatment of heart failure, myocarditis and venous thromboembolic complications, even in cases of thrombocytopenia.

**Table 3:** Comparison of answers given by cardiologists and oncologists regarding general understanding of cardio-oncology in daily practice.

Question	Response Options to the Question	Cardiologists *n* = 52	Oncologists *n* = 52	*p*-Value
What type of cancer do you observe the most common CV complications for?	A. Breast cancer	18 (36.7)	15 (30.6)	*p* = 0.003
	B. Prostate cancer	4 (8.2)	4 (8.2)	
	C. Lung cancer	12 (24.5)	27 (55.1)	
	D. Hematologic cancers	15 (30.6)	3 (6.1)	
The most difficult thing about being a cancer patient is that it makes you…	A. …to diagnose heart failure during oncological treatment (as side effects of the drugs might mimic heart failure symptoms).	5 (9.6)	7 (14)	*p* = 0.45
	B. …to treat thromboembolic complications with DOACs.	8 (15.4)	13 (26)	
	C. …to treat thromboembolic complications in patients with thrombocytopenia.	22 (42.3)	17 (34)	
	D. …diagnose myocarditis in patients treated with immunotherapy.	17 (32.7)	13 (26)	
What cardiologic problem in cancer patients do you observe most often?	A. Heart failure	23 (44.2)	6 (12)	*p* = 0.0002
	B. Atrial fibrillation	9 (17.3)	4 (8)	
	C. Venous thromboembolism	17 (32.7)	38 (76)	
	D. Coronary syndromes	3 (5.8)	2 (4)	
What kind of cardiology consultation are you asked for as cardiologists, or do you ask for as an oncologist?	A. CV assessment before surgery	25 (49.0)	0 (0)	*p* < 0.000001
	B. CV assessment before chemotherapy, immunotherapy, or targeted therapy	14 (27.5)	36 (72)	
	C. CV toxicity assessment	10 (19.6)	12 (24)	
	D. Uncontrolled BP, lipid profile or glycaemia assessment	2 (3.9)	2 (4)	
Do you provide cardiology assessment in cancer patients after their cancer treatments?	A. No, because I do not provide service to such a group of patients.	13 (25)	5 (10)	*p* = 0.000004
	B. I make a cardiology assessment based on anamnesis and physical examination only.	3 (5.8)	24 (48)	
	C. I only control CV risk factors, i.e., BP, lipid profile, glycaemia.	2 (3.9)	5 (10)	
	D. I refer for echocardiogram and electrocardiogram, and I control CV risk factors.	34 (65.4)	16 (32)	

Legend: DOAC—direct oral anticoagulant; CV—cardiovascular; BP—blood pressure.

Interestingly, the most significant clinical issue in cardio-oncology is viewed differently between specialties. Cardiologists most often indicated heart failure (23, 44.2%), while oncologists indicated venous thromboembolism, including pulmonary embolism (38, 76%). Even more surprisingly, the main reason for consultation among cancer patients was perceived differently. Cardiologists most often indicated consultation before surgery (25, 49.0%). Oncologists saw the main reason for consultation in assessing the patient before starting anticancer pharmacotherapy (36, 72%).

There was also a significant difference in defining long-term follow-up after oncological treatment. Cardiologists responded, “I refer for echocardiogram and electrocardiogram, and I control cardiovascular risk factors” (34, 65.4%). In contrast, oncologists stated, “I conduct a cardiology assessment based on the medical history and physical examination only” (24, 48%).

### 3.4. Problematic Clinical Decisions in Cardio-Oncology

The five questions concerned selected difficult clinical decisions in cardio-oncology (Table 4). A significant difference was noted in the responses regarding the recommended treatment for hypertension with blood pressure >160/100 mmHg. Cardiologists most often preferred angiotensin converting enzyme inhibitor or angiotensin receptor blocker (ACEi/ARB) and calcium channel blocker (CCB) (24, 46.2%), while oncologists decided on only ACEi/ARB (16, 32%).

**Table 4:** Comparison of answers given by cardiologists and oncologists, including problematic decisions in cardio-oncology.

Question	Response Options to the Question	Cardiologists *n* = 52	Oncologists *n* = 52	*p*-Value
What do you recommend for cancer patients with BP > 160/100 mmHg?	A. Only ACEi/ARB	2 (3.8)	16 (32)	*p* = 0.0005
	B. ACEi/ARB and CCB	24 (46.2)	9 (18)	
	C. ACEi/ARB and beta-blocker	15 (28.9)	13 (26)	
	D. ACEi/ARB and diuretic	11 (21.2)	12 (24)	
When do you recommend lipid profile tests in cancer patients?	A. Depended on cancer treatment type	5 (9.6)	27 (55.1)	*p* = 0.000003
	B. Only in patients with coronary artery disease	5 (9.6)	3 (6.1)	
	C. Only in patients with lipid disorders	23 (44.2)	16 (32.7)	
	D. Always with cancer treatment initiation	19 (36.5)	3 (6.1)	
What do you recommend for cancer patients with incidentally diagnosed pulmonary embolism?	A. LMWH initiated in outpatient clinic	12 (23.1)	32 (64)	*p* = 0.000001
	B. DOAC initiated in outpatient clinic	21 (40.4)	0 (0)	
	C. UFH/LMWH initiated in inpatient clinic	12 (23.1)	15 (30)	
	D. DOAC initiated in inpatient clinic	7 (13.4)	3 (6)	
When do you recommend LMWH for stroke prevention in cancer patients?	A. When the oncologist recommended	5 (10)	9 (18.8)	*p* < 0.000001
	B. When contraindications for DOAC or VKA occurred	41 (82)	13 (27)	
	C. When brain metastases diagnosed	2(4)	1 (2.1)	
	D. Never	2 (4)	25 (52.1)	
In what clinical situation do you see the need to use antithrombotic prophylaxis for venous thromboembolism?	A. Pancreatic cancer	21 (45.6)	21 (44.7)	*p* = 0.62
	B. Lung cancer	11 (23.9)	7 (14.9)	
	C. When Khorana ≥2	6 (13.1)	7 (14.9)	
	D. When Khorana ≥3	8 (17.4)	12 (25.5)	

Legend: ACEi—angiotensin converting enzyme inhibitor; ARB—angiotensin receptor blocker; CCB—calcium channel blocker; DOAC—direct oral anticoagulant; LMWH—low-molecular-weight heparin; VKA—vitamin K antagonist; UFH—unfractionated heparin.

Another notable issue emerged regarding when to order a lipid profile in cancer patients. Cardiologists ordered it only for patients with lipid disorders (23, 44.2%), while oncologists ordered it depending on the type of cancer treatment (27, 55.1%).

The last three questions referred to anticoagulants. In treating asymptomatic incidental pulmonary embolism, cardiologists most often selected direct oral anticoagulant (DOAC) initiated in an outpatient clinic (21, 40.4%), while oncologists preferred low-molecular-weight heparin (LMWH) initiated in an outpatient clinic (32, 64%). When asked about the use of LMWH for stroke prevention in patients with atrial fibrillation, cardiologists most often indicated the answer “when contraindications for DOAC or vitamin K antagonist (VKA) occurred” (41, 82%). Oncologists, on the other hand, most often stated the answer “never” (25, 52.1%). There were no differences in the perception of indications for primary prevention between cardiologists and oncologists.

### 3.5. The Key Answers of Cardiologists

Among the key answers, two were significantly related exclusively to cardiologists. Cardiologists were almost six times more likely to choose heart failure as the most critical issue in cardio-oncology (OR = 5.82; 95% CI: 2.08–16.22; *p* = 0.0007) (Table 5).

In long-term follow-up, cardiologists consistently recognized the clinical importance of ordering echocardiographic and electrocardiographic tests and managing all cardiovascular risk factors (OR = 4.01; 95% CI: 1.74–9.25; *p* = 0.001).

The second significant opinion was the selection of ACEI/ARB combined with CCB as an option for treating hypertension in oncology, four times more often (OR = 3.9; 95% CI: 1.56–9.75; *p* = 0.003).

The chance that a cardiologist will order a lipid profile is nearly tenfold reduced depending on the type of cancer therapy (OR = 0.09; 95% CI: 0.03–0.26; *p* = 0.000009).

### 3.6. The Key Answers of Oncologists

Among the responses directly concerning the ESC guidelines on cardio-oncology (Table 1), the most important observation was that the fewest oncologists selected the answer “I read the guidelines, but I think they are too complicated to follow”. The odds ratio for an oncologist choosing this response was five times lower compared to cardiologists (OR = 0.21; 95% CI 0.06–0.68; *p* = 0.009) (Table 6).

In terms of general understanding of cardio-oncology, oncologists were almost four times more likely to respond that cardiovascular complications are most frequently observed in lung cancer (OR = 3.78; 95% CI: 1.58–9.05; *p* = 0.002), and they were six and a half times more likely to indicate venous thromboembolism as the most common problem in cardio-oncology (OR = 6.52; 95% CI: 2.7–15.73; *p* = 0.00002).

Oncologists anticipated a cardiovascular evaluation before beginning anticancer treatment (OR = 6.8; 95%: 2.81–16.42; *p* = 0.00002).

Regarding therapeutic decisions, oncologists significantly more often opted for ACEI/ARB monotherapy in the treatment of high blood pressure (OR = 11.76; 95% CI: 2.49–55.54; *p* = 0.002), and significantly more often chose low-molecular-weight heparin in the treatment of asymptomatic incidental pulmonary embolism (OR = 5.93; 95% CI: 2.47–14.24; *p* = 0.00006).

## 4. Discussion

Guidelines created at the interface of different areas of medicine should integrate the work of representatives from these specialties. The ESC guidelines on cardio-oncology are not only dedicated to cardiologists. One of the document’s aims is its implementation by professionals in the field of oncology. The current study shows that this can be challenging if the perception of the main problems in cardio-oncology differs significantly. Our study showed that the clinical experiences and decisions of oncologists are entirely different from those of cardiologists.

Cardio-oncology requires cardiologists to possess a certain degree of knowledge about cancer diseases and their treatment [14]. Being a good general cardiologist, or even a specialist in cardiac imaging, is insufficient to become a competent cardio-oncologist who genuinely comprehends the expectations of oncologists regarding the scope of consultations. It is somewhat surprising in our study that such a large proportion of cardiologists stated that they see consultations before surgical treatment as their most crucial task in cardio-oncology. Cardiologists who answered in this way appear not to have read the table of contents or the first pages of the ESC guidelines on cardio-oncology. The core of the guidelines is to reduce cardiovascular toxicity risk related to various cancer therapies, including chemotherapy, targeted agents, immune therapies, and radiation therapy. Perhaps another reason for such an answer is that these cardiologists do not work in a place with clinical oncology where pharmacological cancer therapy is used in combination with radiation therapy.

The scope of cardiologists’ activities in oncology is significant and discussed in the medical literature [15]. However, the perioperative management of cancer patients is rather the subject of another ESC guidelines document [16]. Oncologists expect cardiology consultations to precede the start of anticancer treatment. It may also be surprising that oncologists were much less likely to assess the ESC document as too complicated. Knowing the realities of their anticancer treatment, oncologists fully understand even the detailed recommendations of the guidelines. The only problem may be the organization of cardio-oncologists’ work, so that consultations take place without unnecessary delay and quick access to a cardiologist experienced in cardio-oncology is ensured [17].

**Figure 1:**
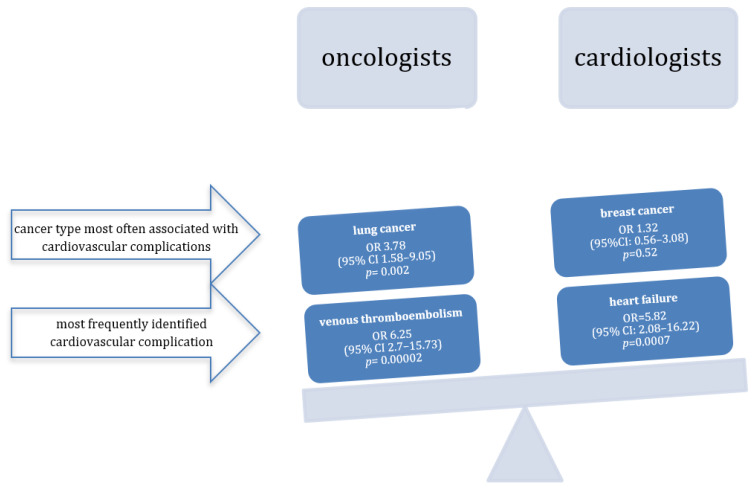
How do oncologists perceive cardio-oncology differently than cardiologists?

Cardiologists see heart failure as the main problem in cardio-oncology (Figure 1). It should be highlighted that the study has been performed by the Polish Society of Clinical Oncology and the Heart Failure Association of the Polish Cardiac Society. First, the simplest explanation of this result is that the cardiologists from the mentioned association could be more interested in heart failure. Additionally, the guidelines dedicated to heart failure, published by the European Society of Cardiology in 2021, were the first in Europe to mention that cardiologists consulting cancer patients must have sufficient experience in cardio-oncology [18]. Looking at everyday practice, it is no surprise heart failure experts are particularly interested in cardio-oncology, as evidenced by recent American and European publications [19,20]. Cardiologists often admit cancer patients to the hospital due to heart failure [21]. Moreover, many cardiologists see cardio-oncology through the prism of breast cancer, where this prevalent cancer in women has already given a lot of evidence of the adverse effects of breast cancer therapy and causes cardiac complications [22]. Many cardiologists also understand the idea of GLS assessment and declare that they fully implement the ESC guidelines into everyday practice. While oncologists still know little about the practical use of GLS, rather than expecting the LVEF measurements result, they already know about the possibility of using classic biomarkers. This is probably due to previous expert documents and the ease of estimating biomarkers, even in such challenging times as the COVID-19 pandemic [23,24].

As expected, cardiologists, based on the new ESC guideline documents, will prefer to treat hypertension with two drugs as combination therapy from the beginning, namely an angiotensin converting enzyme inhibitor or angiotensin receptor antagonist (ACEI/ARB) with cardioprotective properties and a calcium channel blocker (CCB) which acts rapidly and is less likely to cause adverse pharmacological interactions [25]. Although the survey has been completed by cardiologists experienced in the treatment of heart failure, when choosing hypertension therapy, they preferred the combination of ACEi/ARB with CCBs over a beta-blocker as the therapy of choice. Such a decision was consistent with the 2022 ESC guidelines on cardio-oncology. Beta-blockers may have cardioprotective properties in oncology, which was confirmed by randomized trials and real-world data [26,27,28]. However, there is no direct scientific evidence supporting the use of a beta-blocker as the first choice in cancer patients with arterial hypertension.

An international survey performed by the Council of Cardio-Oncology confirmed that cardiologists also prefer new oral anticoagulants (DOACs) in oncology [29]. In stroke prevention for atrial fibrillation, they will decide on low-molecular-weight heparin if there are contraindications to new oral anticoagulants that are not vitamin K antagonists.

It is worth taking a closer look at the key responses of oncologists if partnerships with cardiologists are to become an everyday occurrence. Oncologists usually care for cardiovascular problems among patients with lung cancer (Figure 1). There are three main problems in lung cancer. A large proportion of patients have pre-existing cardiovascular diseases before diagnosis of lung cancer [30]. There is observed a significant impact of anticancer drugs on cardiovascular health in lung cancer [31]. Progression of lung cancer may be associated with abnormalities in cardiac function which can be found on echocardiography [32,33].

Another important response from oncologists is the perception of venous thromboembolism (VTE) as the major problem in cardio-oncology (Figure 1). This opinion may result from the high incidence of VTE observed every day, which is pathophysiologically related to both the development of the cancer itself and its treatment [34,35,36]. The observed impact of VTE on patient mortality may be even more significant [37]. Even with the use of the newest anticancer therapies, there is an association between VTE and significantly shortened survival [38,39].

### 4.1. Therapeutic Challenges

Cardio-oncology includes diagnostic and therapeutic challenges. It is interesting that oncologists, in the case of high blood pressure values, reach for monotherapy with ACEI/ARB. This is probably due to the already established knowledge of the beneficial cardioprotective effect of these drugs on cardiotoxicity [40]. Moreover, there is data that these drugs may provide an additional benefit in prolonging survival during the treatment of hypertension in oncology [41,42].

The next question shows how well-established and consolidated knowledge influences oncologists’ decisions, where oncologists responded that they still prefer to use low-molecular-weight heparin (LMWH) for treating pulmonary embolism, even in asymptomatic episodes. Randomized clinical trials have shown that DOACs are noninferior to LMWH in terms of efficacy in treating VTE in oncology [43]. The problem is safety. The risk of bleeding in gastrointestinal cancers may be increased with some DOACs [44,45]. In addition, oncologists encounter many clinical situations that limit the use of oral anticoagulation. An example is significant thrombocytopenia [46]. Another problem is the concern about reduced liver efficiency where DOACs are metabolized. The additional impact of oncological drugs on the risk of bleeding is also significant [47]. It seems that oncologists’ decisions are based on the established position of LMWH in oncology for many years and the relatively recently implemented recommendations regarding DOAC [48,49].

### 4.2. Diagnostic Challenges

Finally, the significant differences in the perception of diagnostics in cardio-oncology are worth noting. A cardiologist will rarely order a lipid profile assessment depending on the type of anticancer treatment. However, it is crucial that an oncologist uses therapies that do not induce lipid disorders. Failure to assess lipid profile in the case of therapies such as some Anaplastic Lymphoma Kinase (ALK) inhibitors may have very negative consequences [50,51]. After all, oncologists may also use therapies that accelerate atherosclerosis or induce acute coronary syndromes [52]. Evaluation of the lipid profile is also key here. Cardiologists seem to be unaware of this risk associated with the activity of anticancer drugs.

A very positive result is the fact that cardiologists were four times more likely to see sense in ordering echocardiography examinations in long-term follow-up after the completion of anticancer treatment. This could result from the available data of international studies [53,54]. Oncologists based decisions rather only on the patients’ medical history and physical examination during long-term follow up. Such oncologists’ scepticism may result from limited organizational access to cardiology diagnostics tests and a lack of formal financing with confirmation of beneficial cost-effectiveness [55]. ESC guidelines on cardio-oncology highlighted that after the completion of oncological treatment, patients require specialized cardiology supervision during long-term follow-up. The late effect of CTR-CVT may become apparent many years after the completion of treatment and may lead to premature cardiovascular death [56]. When oncologists become more familiar with the ESC guidelines, the situation may be changed.

There was agreement between oncologists and cardiologists on the use of cardiac biomarkers, and this is a result showing that oncologists probably learned about the diagnostic value of biomarkers during their specialization training. The significant differences were obtained in assessing the usefulness of global longitudinal strain (GLS) in echocardiography. This confirms that oncology training did not include knowledge on how to assess left ventricular strain and how to use this parameter in diagnosing cardiotoxicity. It seems reasonable to emphasize that in drug programs and standards addressed to oncologists, LVEF is mainly recommended for assessing heart function. This may be a likely reason for the decreased awareness of the role of GLS in oncology. Further studies are needed to confirm the usefulness of GLS in assessing the indications for cardioprotection and predicting prognosis. Training is needed for oncologists to learn the latest diagnostic possibilities in assessing heart function as part of collaboration models and shared care of cardio-oncology patients.

The picture that emerges from our study is that oncologists with greater awareness of the effects of oncological drugs make therapeutic decisions in cardio-oncology. Less knowledge of the mechanisms of action of various anticancer therapies makes it difficult for cardiologists who mainly rely on ESC guidelines to take targeted actions.

The main question is why cardio-oncology has so many significant interdisciplinary barriers. Oncologists and cardiologists have completely independent education. Cardio-oncology training programs are available but have not been implemented officially in many countries, especially during specialty programs in medical oncology [57,58,59]. Moreover, European oncologists have separate guidelines regarding cardiovascular disorders [23].

A final issue that should be highlighted based on this survey is the difference in priorities between oncologists and cardiologists. Depending on the cancer disease stage, oncologists try to prolong disease-free or progression-free or overall survival. Meanwhile, there is limited evidence on how cardio-oncology can prolong overall survival in cancer patients.

Cardiologists feel obligated to use GLS for early detection of heart dysfunction, optimal control of lipid disorders, and optimal anticoagulation in cancer patients. However, it should be honestly acknowledged that there is currently much controversy surrounding the use of GLS, even in breast cancer patients receiving anthracyclines or anti-HER2 therapy [60]. There are also no dedicated studies demonstrating the effects of treating lipid disorders in oncology, meaning whether it translates into reducing acute coronary events. Similarly, studies related to anticoagulant therapy do not demonstrate an impact on patient survival in oncology.

Therefore, a simple question from oncologists to cardiologists is whether cardio-oncology alone can prevent only severe forms of CTR-CVT and thus prevent premature discontinuation of cancer therapy and finally premature all-cause mortality [61]. Cardio-oncology awaits studies demonstrating that cardiac interventions in oncology will improve overall survival. This trend has been demonstrated, for example, by retrospective study with sodium glucose co-transporter 2 (SGLT2) inhibitors [62,63].

### 4.3. Limitations of the Study

The 104 opinions collected during the study are not significantly different from similar surveys published in prestigious journals [64,65,66]. The problematic issue is that the survey was conducted only in Poland, which limits its value as a regional survey. However, each country has a particular organization of oncology care. In this regard, a survey from one country retains its value, as its results can be cited by physicians from other countries who work in similar or completely different oncology and cardiology care organizations.

The next issue is that each survey is based on voluntary participation, which can lead to potential bias. It should be emphasized that each a survey may not reflect actual practice; the collection of self-reported opinions may be associated with social desirability bias. However, only in this way scientific societies can check a real impact of guidelines on every day activity of different groups of physicians.

The survey omitted important areas highlighted by the ESC guidelines on cardio-oncology like risk stratification tools, multidisciplinary team (MDT) processes, and survivorship pathways. The reason is purely practical. A survey that is too long will not be readily completed by volunteers. Therefore, the survey’s authors, based on their own subjective opinions, decided to ask only about key issues. Moreover, the survey did not ask about ambulatory heart rhythm monitoring. The problem of diagnosing heart rhythm disorders in cardio-oncology is significantly underestimated. Heart rhythm abnormalities may be a significant side effect in cancer patients [5,32]. The 2022 ESC guidelines on cardio-oncology do not focus much on this issue. Recently, a cardio-oncology survey has been published focusing on remote patient monitoring using mobile devices, which is undoubtedly the future [67]. However, this will incur additional costs for the system, and the question is whether it will be available in every cancer centre.

It should be clarified that the survey question “How many cardio-oncology patients do you consult/manage every week?” referred to number of consultations of patients with both cancer and cardiovascular diseases. In our study a high number of the responders had low activity in cardio-oncology. The least experienced cardio-oncology clinicians are those who consult fewer than 10 patients per week. They may see cardio-oncology patients occasionally or not at all. This group of physicians is also unlikely to be interested in deepening their knowledge of cardio-oncology.

### 4.4. Recommendations for the Future

The multitude of mechanisms linking cardiovascular and oncological events is enormous. Most of them require an individual approach, encompassing the specificity of both groups of diseases. To better understand the needs of patients with cancer and the mutual expectations of oncologists and cardiologists, detailed training conducted by experts seems necessary for groups of doctors mainly involved in this group of diseases. As emphasized by the ESC guidelines, critical decisions to discontinue anticancer treatment for cardiological reasons cannot be made by a single person but rather by multidisciplinary teams (MDT) in which a cardio-oncologist should be a member.

There is a need for structural reforms with building implementation strategies of the ESC guidelines in daily practice.

## Figures and Tables

**Table 5:** The odds ratio of essential answers given by cardiologists.

Question	Answer	Cardiologists *n* = 52	Oncologists *n* = 52
What cardiologic problem in cancer patients do you observe most often?	Heart failure	OR = 5.82 95% CI: 2.08–16.22 *p =* 0.0007	ref.
Do you provide cardiology assessment in cancer patients after their cancer treatments?	I refer for echocardiogram and electrocardiogram, and I control CV risk factors.	OR = 4.01 95% CI: 1.74–9.25 *p =* 0.001	ref.
What do you recommend for cancer patients with BP >160/100 mmHg?	ACEi/ARB and CCB	OR = 3.9 95% CI: 1.56–9.75 *p =* 0.003	ref.
When do you recommend lipid profile tests in cancer patients?	Depended on cancer treatment type	OR = 0.09 95% CI: 0.03–0.26 *p =* 0.000009	ref.

ref.—oncologists as reference point for OR calculations.

**Table 6:** The odds ratio of pivotal answers given by oncologists.

Question	Answer	Cardiologists *n* = 52	Oncologists *n* = 52
2022 ESC Guidelines on cardio-oncology.	I read the guidelines, but think they are too complicated to adhere to.	ref.	OR = 0.21 95% CI 0.06–0.68 *p =* 0.009
What type of cancer do you observe the most common CV complications for?	Lung cancer	ref.	OR = 3.78 95% CI: 1.58–9.05 *p =* 0.002
What cardiologic problem in cancer patients do you observe most often?	Venous thromboembolism	ref.	OR = 6.52 95% CI: 2.7–15.73 *p =* 0.00002
What kind of cardiology consultation are you asked for as cardiologists, or do you ask for as an oncologist?	CV assessment before chemotherapy, immunotherapy, or targeted therapy	ref.	OR 6.8 95% 2.81–16.42 *p* = 0.00002
What do you recommend for cancer patients with BP > 160/100 mmHg?	Only ACEi/ARB	ref.	OR = 11.76 95% CI: 2.49–55.54 *p =* 0.002
What do you recommend for cancer patients with incidentally diagnosed pulmonary embolism?	LMWH was initiated in the outpatient clinic	ref.	OR = 5.93 95% CI: 2.47–14.24 *p =* 0.00006

ref.—cardiologists as reference point for OR calculations.

## Data Availability

The datasets used and/or analyzed during the current study are available from the corresponding author on reasonable request.
